# Differentiation of mouse bone marrow derived stem cells toward microglia-like cells

**DOI:** 10.1186/1471-2121-12-35

**Published:** 2011-08-19

**Authors:** Arnd Hinze, Alexandra Stolzing

**Affiliations:** 1 Fraunhofer Institute for Cell Therapy and Immunology (IZI), Perlickstrasse 1, 04103, Leipzig, Germany

**Keywords:** bone marrow stem cells, microglia, Flt3L, GM-CSF, neurodegeneration, differentiation

## Abstract

**Background:**

Microglia, the macrophages of the brain, have been implicated in the causes of neurodegenerative diseases and display a loss of function during aging. Throughout life, microglia are replenished by limited proliferation of resident microglial cells. Replenishment by bone marrow-derived progenitor cells is still under debate. In this context, we investigated the differentiation of mouse microglia from bone marrow (BM) stem cells. Furthermore, we looked at the effects of FMS-like tyrosine kinase 3 ligand (Flt3L), astrocyte-conditioned medium (ACM) and GM-CSF on the differentiation to microglia-like cells.

**Methods:**

We assessed *in vitro-*derived microglia differentiation by marker expression (CD11b/CD45, F4/80), but also for the first time for functional performance (phagocytosis, oxidative burst) and *in situ *migration into living brain tissue. Integration, survival and migration were assessed in organotypic brain slices.

**Results:**

The cells differentiated from mouse BM show function, markers and morphology of primary microglia and migrate into living brain tissue. Flt3L displays a negative effect on differentiation while GM-CSF enhances differentiation.

**Conclusion:**

We conclude that *in vitro-*derived microglia are the phenotypic and functional equivalents to primary microglia and could be used in cell therapy.

## Background

Microglias constitute about 10% of the cell population of the brain and represent the most important first immune defense of the CNS. They are phagocytic, cytotoxic, antigen-presenting cells which promote brain tissue repair after injury [1]. Primary microglia differ from other blood macrophages in the expression levels of markers like CD11b/CD45low/high [2], CD68 low/high [3] and substance P levels [4]. Because of the overlap in markers there is an ongoing discussion about the distinction between dendritic cells, macrophages and microglia. The regulation of marker levels and activity has led to the proposition that microglia could be immature or resting macrophages [5]. However, there is a lack of correlation between marker expression and actual functional capacity, which is the most important hallmark for therapeutic use. Microglia in the brain normally display a quiescent state in which phagocytosis, immune response and migration are down-regulated and the microglia show a ramified morphology with long processes [6]. Microglia react to inflammation by switching to an activated state and taking on an amoeboid morphology [7]. They migrate towards sites of injury and lesion and extracellular debris such as amyloid-β plaques [8]. An important function of microglia is the "oxidative burst" - a sudden spike in reactive oxygen species (ROS) levels generated by the stimulation of the NADPH oxidase. This ROS production is accompanied by the release of other factors, including lysosomal proteases. This mechanism, often interpreted as a 'defense' response that can protect the brain from pathogens, is a characteristic feature of microglia [9,10]. Microglia are thought to originate from the yolk sac during embryogenesis [11] and are replenished by local proliferation throughout adult life. The supplementation by progenitor cells from the bone marrow is controversial [1,11,12]. Bone marrow-derived microglia can be observed in the brain after systemic transplantation [13]. While BM chimeras have shown BM-derived microglia [14], other findings indicate that without irradiation no invasion is observable in the time frame of 1-2 months [15,16]. But also in transplantations without irradiation intravenously injected hematopoietic stem cells have been observed to migrate to the brain, differentiate into microglia and reduce infarct size [17]. The maturation of progenitors to microglia occurs under the influence of factors secreted by astrocytes [18]. Both local and peripheral replenishment do not seem to suffice to prevent the slow deterioration of the microglia cell population and function with age [19,20]. In human Alzheimer patients microglia associated with tau tangles were found to be dystrophic, which might precede neurodegeneration [21]. In old rats there have been indications that the proliferation of microglia after injury is stronger than in young rats [22]. *In vitro*, proliferating rat microglia have been reported to undergo telomere shortening [23] and aged microglia of several species have been observed to loose their ability to perform normal microglia functions [19,20,24-28]. These findings support the hypothesis of a slow deterioration of microglia as a contribution to the onset of neurodegeneration [20,21].

The maturation of progenitors to microglia occurs under the influence of factors secreted by astrocytes [16]. Both local and peripheral replenishment do not seem to suffice to prevent the slow deterioration of microglia cell population and function with age [17,18]. The resident microglia are suspected to reach replicative senescence during aging [18]. Microglia have been differentiated *in vitro *from peripheral blood monocytes [4,18] and from embryonic stem cells [29]. In this context, we focus on differentiating microglia from bone marrow. This approach was first demonstrated by Servet-Delprat et al. [30], who obtained 20% cells with microglia-like morphology and marker expression (CD115+, CD11b+, F4/80+, CD80 low, CD86-) after culturing mouse BM cells in Flt3L for 11 days and then mixing the cell-containing supernatant with astrocyte-conditioned medium for 6 days. However, since the use of Flt3L was not controlled in that protocol, its role as a factor in microglia differentiation remained unclear. Davoust et al. [31] used a similar protocol but significantly shorter culture times and no Flt3L to obtain CD11b +, CD45 +, MHCII -, B220 low, CD34+, and CD86 low cells from mouse BM (the percentage yield is not reported). The success of microglial cell differentiation has been mostly judged by measurement of the expression of markers and the morphology of the differentiated cells. It remained unclear to what extent *in vitro-*derived microglia-like cells share the functional capacities of original microglia. To address this question, we followed the protocol of Servet-Delprat et al. [30] (with and without Flt3L), measured phagocytosis and oxidative burst as hallmarks of microglial function and tested the ability to survive and migrate in brain tissue.

## Results

### Surface marker expression

Untreated bone marrow cells showed significantly increased CD11b/CD45 expression after 17 days in culture. The same was observed in cultures treated with ACM/GM-CSF. Non-adherent BM cells treated with ACM/GM-CSF and whole bone marrow and cultivated for 17 days are observed in the same region as primary microglia in the flow cytometry plots. Flt3L has an adverse effect on differentiation, leading to low levels of CD11b/CD45-positive cells in all Flt3L-supplemented samples.

**Figure 1:**
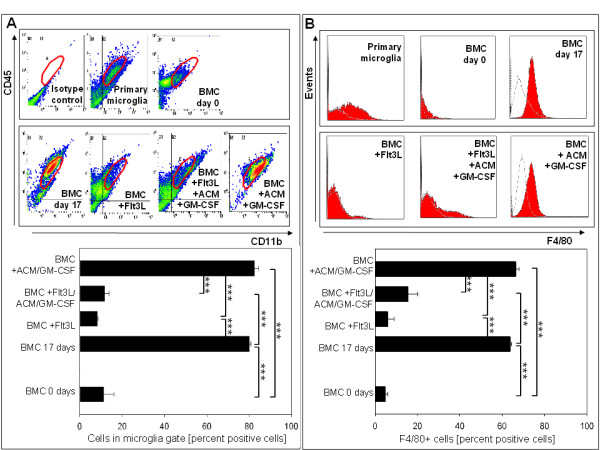
**Flow cytometric analysis of adherent? BM cells**. **(A) **Flow cytometric analysis of adherent? BM cells on CD11b/CD45 expression (n = 3) following culture in vitro for certain period of time. Representative scatter plots of CD11b/CD45 labeled differentiated cells and primary microglia, fresh bone marrow and an isotype control. **(B) **Flow cytometric analysis of adherent? BM cells on their F4/80 expression (n = 3). Representative histogram plots of F4/80 labeled differentiated cells, primary microglia and fresh bone marrow. Isotype control gray, F4/80 labeled cells black. *** = P < 0.001, ** = P < 0.01, * = P < 0.05.

The frequency of F4/80+ cells already increased significantly in whole bone marrow after 7 days and also after 17 days, as compared to fresh bone marrow. The supplementation of Flt3L or Flt3L/ACM/GM-CSF resulted in significantly lower F4/80+ cell numbers while the sole addition of ACM/GM-CSF yielded high numbers of F4/80+ cells very similar to the CD11b/CD45 cell populations (Figure 1, Table 1).

**Table 1:** Flow cytometric analysis of the cells differentiated and analyzed for microglia specific markers

Protocol	Supplementation	CD11b percent	CD11b median	F4/80 percent	F4/80 median	CD11b/CD45 percent	Cells in microglia gate
**BM 0 days**		**20**+/-9.5	**2**+/-0.7	**2.3**+/-0.1	**1.2**+/-0.1	**20**+/-9.5	**6,7**+/-3,6

**BM 7 days**		**51,2**+/-15,5	**7,8**+/-3,5	**55,9**+/-5,3	**4,4**+/-0,6	**54,7**+/-15,0	**54**+/-19,7

**BM 10 days**		**67,3**+/-2,8	**8,8**+/-1,5	**59,8**+/-3,3	**4,3**+/-0,3	**72,8**+/-2,9	**70,3**+/-4,1

**BM 17 days**		**68,4**+/-0,6	**13,8**+/-0,4	**64,5**+-0,6	**3,6**+/-0,1	**70**+/-0,5	**84,2**+/-0,5

**Sup. BM day 11**		**71,7**+/-3,6	**9,5**+/-1,3	**40,5**+/-5,1	**2,7**+/-0,4	**71,7**+/-3,6	**62,7**+/-6,5

**Sup. BM day 11**	Flt3L	**35,8**+/-12,9	**3,6**+/-2,1	**6,8**+/-1,3%	**1**+/-0,2	**35,8**+/-12,9	**20,1**+/-18,8

**Protocol 2**	ACM/GM-CSF	**85,6**+/-1,5	**23,3**+/-2,9	**66,4**+/-1,7	**3**+/-0,3	**89,1**+/-1,5	**90**+/-1,7

**Protocol 3**	Flt3L/ACM/GM-CSF	**39,1**+/-8,1	**2**+/-0,3	**18,8**+/-3,1	**1,3**+/-0,2	**46,6**+/-8,0	**9,9**+/-2,7

**Protocol 4**	Flt3L	**8,2**+/-4,1	**1**+/-0,0	**10,7**+/-5,1	**1,1**+/-0,1	**15,5**+/-4,1	**5,1**+/-0,5

### Time course of marker expression

**Figure 2:**
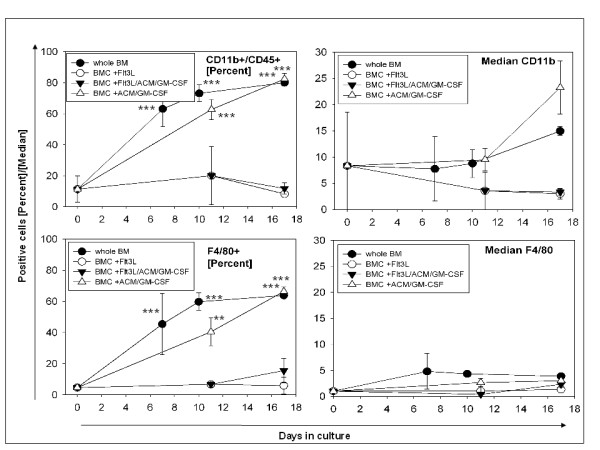
**Time course of CD11b/CD45 and F4/80 expression of untreated and cytokine treated BMC (n = 3)**. Time course of CD11b and F4/80 medians. Significant changes are denoted with respect to freshly isolated BMC on day 0. *** = P < 0.001, ** = P < 0.01, * = P < 0.05.

The frequency of CD11b+/CD45+ and F4/80+ cells in whole bone marrow rose steadily and significantly with extension of culture time (Figure 2, Table 1). The supplementation of ACM/GMCSF to the non-adherent BM cells increased CD11b+/CD45+ and F4/80+ cells over time; however, the increase was significantly slower and only reached the same level as un-supplemented bone marrow at day 17 (it therefore seems that ACM/GM does not have an improvement effect). The supplementation of Flt3L resulted in a lower frequency of CD11b+/CD45+ and also F4/80+ (Figure 2, Table 1). The addition of ACM/GMCSF significantly increased the medians of CD11b expression after 10 days (Figure 2, Table 1).

### Phagocytic activity and oxidative burst

**Figure 3:**
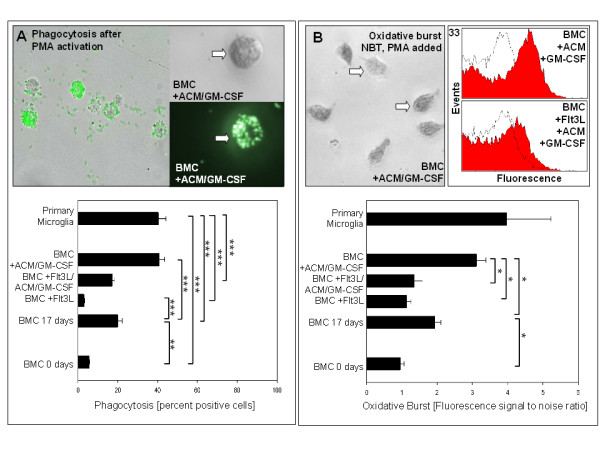
**Phagocytosis and Oxidative burst of differentiated cells**. **(A) **Phagocytosis of differentiated cells and fluorescence microscope picture of phagocytosis of non adherent BM cells differentiated with ACM/GM-CSF (n = 3). Arrows indicate the same cell in bright field and fluorescence picture. Fluorescence images were taken with a Zeiss Axio Observer at 200× (left) and 400× (right) magnification. **(B) **Oxidative burst of differentiated cells, representative histogram plot of inactive (open histogram) and PMA activated (red histogram) cells (n = 3). The shift between un-treated and PMA treated microglia was measured as quotient between medians of treated and untreated cells (Fluorescence signal noise ratio - FSN). Cells with no shift are based at 1 and higher numbers represent cell populations which did show ROS production. Light microscope picture of NBT reduction of non adherent BM cells supplemented with ACM/GM-CSF. Arrows indicate one cell with dark blue NBT precipitate and one cell without precipitate. The picture was taken with a Leica DM IL at 20× magnification. *** = P < 0.001, ** = P < 0.01, * = P < 0.05.

Whole bone marrow cultured over a period of 7, 10 or 17 days showed a constant significantly higher percentage of phagocytic cells compared to fresh bone marrow (Figure 3A). Supplementation with ACM/GM-CSF increased the number of phagocytic cells significantly compared to unsupplemented bone marrow at day 17. Flt3L supplementation significantly inhibited the differentiation towards phagocytizing microglia, even when ACM/GM-CSF was added. We observed the same changes in the amount of microglia performing oxidative burst (Figure 3b); however, the differences were less pronounced.

### Cell morphology

**Figure 4:**
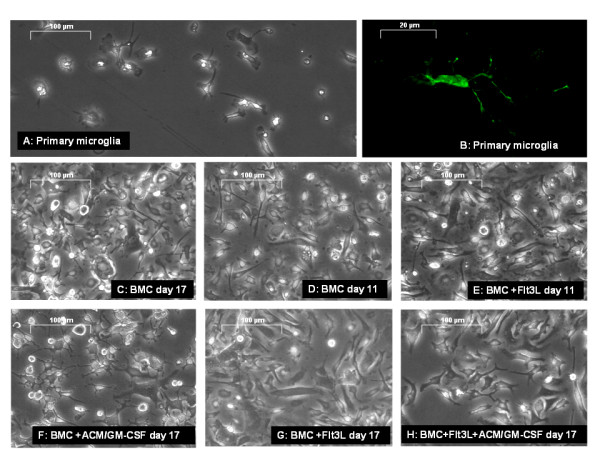
**Representative light microscope pictures of differentiated cells**. Images were taken with a Leica DM IL at 200× magnification. BMC: Whole bone marrow cells. Fluorescence picture of an Iba-1 stained microglia in a brain slice **(B) **was taken with an Axio Imager A1 (Zeiss) at 63× magnification.

Primary microglia show long processes and rod-shaped cells (Figure 4A). Unsupplemented bone marrow cells had mixed morphologies during the whole cultivation time (Figure 4C, D). The cells supplemented with ACM/GM-CSF are more homogenous and show high ramification (Figure 4F). Cells treated with Flt3L alone or in the presence of Flt3L and ACM/GM-CSF both have a more fibroblastic morphology with no resemblance to microglia (Figure 4H).

### Migration in organotypic brain slices

**Figure 5:**
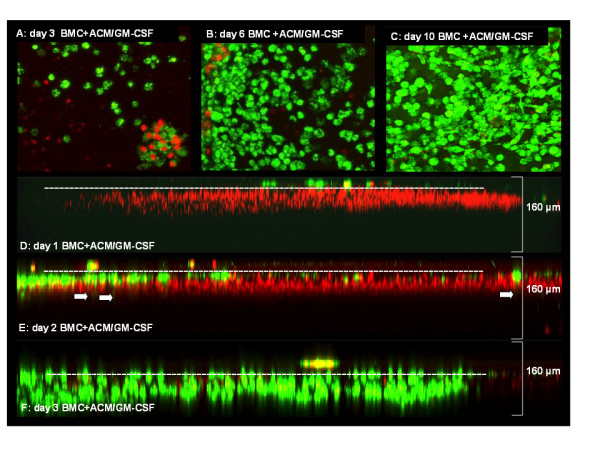
**Coculture with living brain slices**. Differentiated ACM/GM-CSF treated BMC were labeled with DIO and seeded on brain slices on day 9. Counterstaining with propidium iodide was used to assess cell survival. After 1, 2, 3, 6 and 10 days slices were scanned with a Leica Microsystems TCS SP2 confocal microscope to assess survival of seeded cells and their migration into the tissue. Arrows indicate single cells that have already migrated through the surface after 2 days. Magnification was 100x and scanning depth was 160 μm.

**Figure 6:**
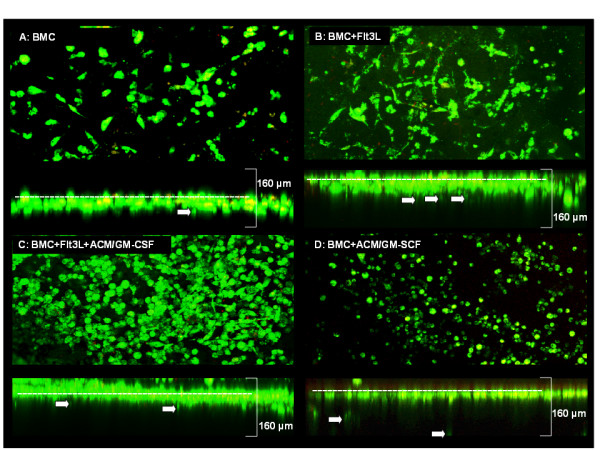
**Differentiated cells of all protocols were labeled with DIO and seeded on brain slices on day 9**. After 10 days coculture slices were propidium iodide stained and scanned with a Leica Microsystems TCS SP2 confocal microscope to measure cell survival and their migration. Arrows indicate several cells that have migrated deeper into the brain tissue. The images were taken at 100× magnification and the brain slices were scanned to a depth of 160 μm.

Whole brain slices were cultured for 10 days to minimize surface damage before differentiated microglia pre-labeled with 3,3'-dioctadecyloxacarbocyanine perchlorate (DiO) were added on top of the brain slices. The slice was counterstained with propidium iodide (PI) to visualize dead cells. Reconstructed confocal images were either top-down or showing a lateral view of the slice. Microglia were observed over a period of 10 days and found to survive and proliferate (Figure 5A-C, dead cells deliberately included for reference). Over the course of 3 days, ACM/GM-CSF-supplemented BMC migrated into the surface of the brain slices as deep as 50 μm (Figure 5D-F). Cells of all protocols migrated up to 30 μm into the slice after 10 days while dead cells stayed on top of the tissue (Figure 6A-D). Several cells migrated up to 120 μm (160 μm confocal microscope scan depth). Cells supplemented with Flt3L have the same fibroblastic morphology as in the *in vitro *cultures (Figure 6B) while additional supplementation with ACM/GM-CSF resulted in round and amoeboid cells as well as fibroblastic cells (Figure 6C). Cells supplemented solely with ACM/GM-CSF almost exclusively showed round cell morphology (Figure 6D). Cells of whole bone marrow were round but larger than the ACM/GM-CSF-supplemented cells after 7 days and did not migrate more than 30 μm into the surface of the brain tissue (Figure 6A).

## Discussion

We investigated the differentiation and function of microglia from bone marrow (BM) stem cells using ACM and GM-CSF with and without Flt3L. As opposed to M-CSF used by Davoust et al. [31], we used GM-CSF as this is reported to expand primary microglia more successfully than M-CSF [32,33]. Primary microglia have been characterized as CD11b+/CD45low and distinguished from primary macrophages on the basis of their CD45 expression level [2]. The *in vitro-*differentiated microglia derived in this manner generally show marker expression levels similar to those of primary microglia. It is known that ACM treatment of BM cells can produce cells with markers for microglia [30]. However, such cells have not been further characterized with respect to phagocytic capacity and migration behavior inside the brain or tested for the microglia-typical oxidative burst. Here we demonstrate that BMC cultured in the presence of ACM and GM-CSF show phagocytosis and oxidative burst activity typical of microglia. The cells also had long and branched processes similar to primary microglia. Flt3L supplementation diminished the functional markers and microglia-like morphology. Thus, among the parameters tested here, the 'optimal' protocol for *in vitro *differentiation of microglia relies on ACM, GM-CSF without Flt3L. Interestingly, we find that even unsupplemented BM contains a subpopulation positive for microglial markers (CD11b/CD45, F4/80) and that this population is more dominant after 17 days of differentiation. However, we find that microglia-like cells derived from BM without any supplementation display only low phagocytosis and oxidative burst levels compared to ACM/GM-CSF-supplemented cells. Generally, unsupplemented bone marrow cultures show mixed cell morphologies whereas supplemented cultures are prone to display more homogeneous, branched cell types. Flt3L has been used for the sequential differentiation of BM cells presumably because it improves hematopoietic stem cell (HSC) survival *in vitro *[30] and *in vivo *[34]. Servet-Delprat et al. only investigated Flt3L-supplemented cells and did not consider unsupplemented cells. The group estimated 20% microglia from the number of ramified cells, which is confirmed by our results for ACM, GM-CSF, Flt3L-supplemented cells. However, much higher functional microglia 'yield' can be obtained in the absence of Flt3L. In fact, we demonstrate that supplementation with Flt3L diminishes microglia differentiation: where Flt3L is added alone or in combination with ACM, GM-CSF, the number of cells showing microglial markers as well as the capacity for brain migration, phagocytosis and oxidative burst decreases. The differentiation protocols investigated here rely on using the supernatant at day 11 to select for non-adherent HSC and then culturing it in the presence of ACM for another 6 days. The tactic here is to first obtain a relatively pure HSC population which then partially differentiates into adherent microglia. Flt3L has been shown to expand HSC and transiently increase adhesion of HSC in culture and it might play a role in mobilization of HSC into the blood stream [35]. Therefore, the amount of microglia progenitor cells in the day 11 supernatant bone marrow culture might be decreased or the differentiation might be delayed. In addition, Flt3L combined with GM-CSF has been shown to enhance dendritic cell differentiation [36]. This fact is supported by work with Flt3L knockout mice where levels of dendritic cells are increased and numbers of myeloid cells, the progenitors of microglia, are decreased [37]. These factors may explain why Flt3L supplementation yields a lower count in functional *in vitro*-derived microglia.

The microglial cell population is known to be heterogeneous and to overlap with dendritic cell-like populations in the brain [38]. The various procedures employed for microglial differentiation might result in distinct subpopulations or activation states. The choice of the protocol might have a substantial impact on the effect transplanted cells will have *in vivo*. This is especially important because different subsets of microglia have been linked to tolerance induction or immune reaction [39].

In co-cultures with organotypic brain slices the microglia-like cells survived and proliferated for at least 10 days. It is known that the majority of primary microglia or BV2 cells only migrate over the surface layer of brain tissues under non-inflammatory conditions [40,41] while a subpopulation migrates into the tissue. Directed migration towards sites of injury induced by NMDA on the surface of brain slice cultures has been observed for primary microglia [41]. The damaged surface of the brain slice cultures even attracts slice-internal microglia, which showed directed migration to the surface [40]. This is supported by our results: Most cells migrate into the brain slice tissue superficially while *in vitro-*derived (ACM/GM-CSF, but without Flt3L) microglia migrated deepest into the tissue and showed both amoeboid and rounded morphologies suggesting an activated state.

The microglial cell population is known to overlap with dendritic cell-like populations in the brain [38]. Dendritic cells differentiate from monocytes and mature by exposure to antigens or under inflammatory conditions [38]. There is evidence that cells showing an immature dendritic phenotype can differentiate from microglia under the influence of GM-CSF [38]. At the same time, dendritic cells can be differentiated to microglia-like cells which inhibit T cell proliferation induced by mature dendritic cells [42]. In the current study, microglia were differentiated using ACM and GM-CSF. There is evidence that cells showing an immature dendritic phenotype can differentiate from microglia under the influence of GM-CSF [38]. At the same time, dendritic cells can be differentiated to microglia like cells which inhibit T cell proliferation induced by mature dendritic cells [42]. Dendritic cells can act both tolerogenic and immunogenic, depending on their maturation state [43]. CD11c-positive microglia have been observed to acquire immature dendritic cell phenotypes in models of acute experimental autoimmune encephalomyelitis (EAE) and to be part of the antigen-presenting cells responsible for the disease [5]. The risk in using *in vitro-*differentiated microglia in a therapy is that these cell populations might contain a dendritic cell-like subpopulation expressing CD11c, which can stimulate an autoimmune inflammation within the CNS [44]. This might lead to the development of an autoimmune phenotype.

It was one of our reasons for performing a functional analysis on our *in vitro-*differentiated microglia that it was described in the literature that the microglial subpopulations displaying similarities to dendritic cells and expressing CD11c (and other dendritic markers) do not contain any phagocytic vacuoles [38]. Our cells are selected for a highly phagocytic activity. This is an additional feature required to potentially clear protein aggregates like amyloid plaques from the brain parenchyma, but would also avoid the transplantation of autoimmune-inducing microglia. A risk still persists, as it was shown that CD11b-positive microglia can produce cells with dendritic features which can acquire the antigen-presenting activities after activation [44]. However, dendritic cells can act both immunogenic and tolerogenic, depending on their maturation state [43]. This could be beneficial for reducing transplant rejection [43] or used to treat autoimmune inflammation, for example in acute experimental autoimmune encephalomyelitis [5,45]. Transplantation of human microglia in ischemic brains modulates inflammation and reduces neuronal apoptosis [46]. Microglia provide neuroprotection in hippocampal slice cultures while lipopolysaccharide-stimulated microglia do not [47]. The various procedures employed for microglial differentiation might result in distinct activation or differentiation states. The choice of the protocol and the composition of the cells might have a substantial impact on the effect transplanted cells will have *in vivo*.

## Conclusion

The *in vitro-*differentiated cells correspond to primary microglia in phenotype and function. The importance of microglia in degenerative diseases makes them an interesting target for therapeutic approaches. If neurodegenerative diseases occur in part due to the age-dependent deterioration of the microglial cell population number and/or function, functional microglia supplementation could have beneficial effects. For example, injection of primary microglia into the brain of rats led to an increased amyloid beta clearance [48]. Furthermore, the suspected ability of microglial precursors to cross the blood-brain barrier and to seek out sites of neuroinflammation renders them potentially useful drug delivery vehicles [49]. *In vitro-*derived microglia will need to demonstrate the functional capacity of 'real' microglial cells and our research makes some contributions to this aim. However, extensive further tests will be required before such cells are deemed suitable and safe for transplantation.

## Methods

### Animals

C57BL/6 mice from the MEZ of the University of Leipzig and Charles River (Sulzfeld, Germany) were used as sources for bone marrow, primary microglia and organotypic brain slices in accordance with local animal ethics permissions.

### Isolation of bone marrow and cell culture

Bone marrow was obtained by centrifugation of femora and tibiae. Isolated bone marrow cells were cultured at a density of 10^7 ^cells in a 60 mm petri dish in 5 ml of Dulbecco's minimal essential medium (DMEM)/low glucose (Hyclone Laboratories Inc.), supplemented with 10% fetal calf serum (FCS -Invitrogen) and 100 units/ml Penicillin, 100 μg/ml Streptomycin.

### Astrocyte-conditioned medium

Astrocyte-conditioned medium was produced by incubating medium (DMEM/10% FCS) for 24 h with primary mouse astrocyte cultures [16].

### Isolation of primary microglia

Primary microglia were isolated from brains of 1-3 day old mice. The meninges was removed and the whole brain was titrated in DMEM/10% FCS and Pen/Strep. The resulting cell and tissue suspension of 3 brains was cultured in a poly L-lysine-coated culture flask. After 24 h, the supernatant was removed from the cell culture and new medium was added. After 7 days, 50% of the culture medium was changed. At 14 days, microglia were removed by gentle shaking [50].

### Differentiation towards microglia-like cells

#### Experiment set 1

Whole bone marrow (10^7 ^cells) was cultivated over the time periods of 7, 10 and 17 days in 10 ml DMEM/10% FCS in a 60 mm petri dish and analyzed for certain cell surface markers at these time points. When cells were cultured for longer than 10 days, 50% of medium was replaced at day 10.

#### Experiment set 2

Whole bone marrow (10^7 ^cells) was cultured for 11 days in a 90 mm petri dish in either plain DMEM/10% FCS or DMEM/10% FCS supplemented with 5 ng/ml Flt3L (noFlt3L and Flt3L groups, respectively). After 11 days, cells of both groups (with and without Flt3L) were analyzed for surface markers and non-adherent cells were further cultured for a period of 6 days. To this end, cells from 2 petri dishes were aspirated, transferred to a new 60 mm petri dish and cultured in DMEM/10% FCS supplemented with 50% ACM and 20 ng/ml GM-CSF (noFlt3L and Flt3L + suppl., groups); or supplements were omitted for Flt3L group (Flt3L - suppl. group).

### Flow cytometry

The differentiated cells were tested for the surface markers F4/80, CD11b, CD45 and CD11b/CD45 double expression. The cells were trypsinized, centrifuged at 300 g for 5 min and fixed in 4% paraformaldehyde. They were washed with phosphate buffered saline (PBS). Afterwards, cells were incubated for 2 h at 4°C with CD11b (1:250) or F4/80 (1:250) antibody (both Alexa 488-labeled, eBioscience) or with CD45 (1:100) antibody (PE-labeled, eBioscience). The incubated cells were washed again and fluorescence was measured with a Beckmann Coulter FC 500.

### Phagocytosis

Phagocytic activity of the differentiated cells was measured by the uptake of fluorescent beads (Sigma, 2 μm yellow/green fluorescent). In a first step, samples of 3*10^5 ^cells were activated with phorbol-12-myristate-13-acetate (PMA) (0.1 μM) for 15 min at 37°C [51]. Afterwards they were incubated in 50 μl DMEM/10% FCS together with 50 μl opsonized (FCS) beads for 48 h at 37°C, 5% CO_2_. The uptake of fluorescent beads was observed qualitatively in a Zeiss Axio Observer fluorescence microscope. For quantitative assessment the cells were trypsinized and resuspended in PBS (Invitrogen). Cells were repeatedly washed and fluorescence was measured in a Beckmann Coulter FC 500.

### Oxidative burst

#### Nitro Blue Tetrazolium (NBT)

10^4 ^cells were seeded on cover slips. They were incubated with 30 μl 1 mg/ml NBT and 100 nM PMA for 45 min at 37°C and 5% CO_2 _[52]. Light microscope pictures were taken with a Leica DM IL (Leica) using the LAZ EZ 1.4.0 software (Leica). Pictures were brightness- and contrast-adjusted with GIMP 2.4.5 and Power Point (Microsoft).

#### Dihydrorhodamine 123 (DHR123)

3*10^5 ^Cells were incubated in PBS for 15 min at 37°C with 0.1 μM PMA. Controls were incubated without PMA. Afterwards, 50 μM DHR123 (Invitrogen) was added and the cells were incubated for additional 15 min at 37°C. The cells were fixed with 4% PFA and fluorescence was measured in a Beckmann Coulter FC 500.

### Cell Morphology

A DM IL (Leica) and the LAZ EZ 1.4.0 software (Leica) were used to take light microscopic pictures.

### Brain slice cultures

2-3 month old C57BL/6 mice were killed by cervical dislocation. The brain was isolated and cut into 350 μm slices in cold preparation medium (HBSS and 10% FCS (Invitrogen) using a Leica VT 1000 S vibratome). The slices were transferred to an insert (Millicell CM 0.4 μm, Millipore) and cultivated with brain slice culture medium (50% DMEM/high glucose (HyClone Laboratories Inc.), 25% Horse Serum (Invitrogen), 25% HBSS (Invitrogen), 1 μg/ml insulin, 100 units/ml penicillin, 100 μg/ml streptomycin). Medium was changed every 2-3 days. The brain slices were cultured for 9 days before cells were seeded on them [53].

### Survival and migration in brain slice cultures

Differentiated cells were labeled with DIO (Invitrogen) for 20 min. They were added on top of the brain slices. The viability of brain slice cultures was checked by performing PI staining. The survival of seeded cells was checked by adding 5 μg/ml propidium iodide to the medium, washing and scanning the slices with a confocal microscope TCS SP2 (Leica Microsystems) using the accompanying software LCS 2.6 (Leica Microsystems). The slices were scanned to a depth of 160 μm after 10 days of co-culture. Images were contrast- and brightness-adjusted with GIMP 2.4.5 and Power Point (Microsoft).

### Statistical Analysis

All data are presented as means ± SE. Statistic analysis were made using SigmaPlot 10.0/SigmaStat 3.5 software (SYSTAT, Erkrath, Germany).

## Competing interests

The authors declare that they have no competing interests.

## Authors' contributions

AH carried out all experiments and wrote the manuscript. AS designed & coordinated the study and contributed to writing the manuscript. All authors read and approved the final manuscript.
